# Supplementary material for: MetaRibo-Seq measures translation in microbiomes
Source: Nat Commun. 2020 Jun 29;11:3268. doi: 10.1038/s41467-020-17081-z (PMC7324362; doi:10.1038/s41467-020-17081-z)
Supplement: Supplementary file 10 — Supplementary Data 7 [file 41467_2020_17081_MOESM10_ESM.zip › File2/Confidence_High_Taxonomy/143168_out.krona.html]

Javascript must be enabled to view this page.

members
magnitude
magnitudeUnassigned
count
unassigned
taxon
rank

143168\_out

3

2
superkingdom
1

1
phylum
976

200643
1
class

order
1
171549

171552
family
1

838
1
genus

species
1

SRS893232\_contig\_number\_22724
28135

superkingdom
2
2759

kingdom
2
4751

451864
subkingdom
2

4890
2
phylum

subphylum
2
147538

147545
class
2

subclass
2
451870

34395
2
order

43219
family
2

5583
genus
2


SRS051031\_contig\_number\_30134SRS098571\_contig\_number\_18734
91920
2
species
